# Understanding science teachers’ implementations of integrated STEM curricular units through a phenomenological multiple case study

**DOI:** 10.1186/s40594-018-0101-z

**Published:** 2018-02-13

**Authors:** Emily A. Dare, Joshua A. Ellis, Gillian H. Roehrig

**Affiliations:** 1 Michigan Technological University, 1400 Townsend Drive, Houghton, MI 49931 USA; 2 University of Minnesota, 1954 Buford Ave, St. Paul, MN 55108 USA

**Keywords:** STEM education, Middle school, Physical science, Engineering, Phenomenology

## Abstract

**Background:**

Current reforms in K-12 STEM education call for integration between science, technology, engineering, and mathematics (STEM). Such integration of STEM disciplines at the K-12 level offers students an opportunity to experience learning in real-world, multidisciplinary contexts; however, there is little reported research about teachers’ experiences in engaging in integrated STEM instruction. The purpose of this phenomenological multiple case study is to understand nine science teachers’ first-time experiences in implementing integrated STEM curricular units in their middle school physical science classrooms. This study draws upon both classroom implementation data and teacher reflective interviews to illustrate different degrees of integrated STEM instruction and to understand teachers’ challenges and successes with these varying approaches.

**Results:**

Our results indicate three distinct cases of integration within our sample that represent low, medium, and high degrees of STEM integration throughout curriculum implementations. Interviews with teachers from each case revealed three themes that varied across teachers’ experiences: the nature of integration, choosing between science and engineering, and student engagement and motivation. Teachers in all three cases were challenged to make explicit connections between science, engineering, and mathematics while simultaneously maintaining a motivating and engaging context for their students throughout their instruction. Further, it appears that the degree of STEM integration that occurs in instruction may be related to teachers’ ability to make explicit connections between the disciplines.

**Conclusions:**

The work presented here informs educational researchers, policy makers, and K-12 STEM educators that there are several challenges when it comes to implementing new STEM initiatives in K-12 education. Although this work is limited to middle school physical science teachers’ experiences with first-time STEM instruction, many of the identified themes are not content-specific; therefore, this work may shed light on general struggles that are common to educators who are integrating across content disciplines for the first time.

## Background

Current national documents call for improvements in K-12 science, technology, engineering, and mathematics (STEM) education to increase STEM literacy and motivate students to pursue careers in these fields (National Academy of Sciences, National Academy of Engineering, & Institute of Medicine of the National Academies, 2007; National Research Council [NRC], 2009, 2012). Others have called for changes in K-12 science education to provide more authentic learning environments for students (National Academy of Sciences, 2014). Specifically, the *Next Generation Science Standards* (NGSS Lead States, 2013) provide guidance for improving K-12 education through the integration of computational thinking and engineering standards in science instruction. These and other adopted standards and curricula also encourage the incorporation of mathematics and technology in STEM education (National Governors Association Center for Best Practices & Council of Chief State School Officers, 2010). It is clear that there is a shift in the way science education is being conceptualized, and such integration of STEM disciplines at the K-12 level offers students an opportunity to experience learning in a real-world, multidisciplinary context. Although the idea of integrated STEM is present in these national documents, the STEM fields are in practice most often taught as isolated disciplines. This current approach does not reflect the natural interconnectedness between disciplines, which has consequences for student interest, knowledge, and performance (Moore et al, 2014).

Despite the push for integrated STEM in K-12 education, there remains a general lack of opportunities for teachers to participate in integrated STEM-related professional development, and existing curricula are not currently designed to support teachers’ integration efforts (English, 2016; National Academy of Engineering, 2009; Roehrig, Wang, Moore, & Park, 2012). Currently, science teachers lack an understanding of the nature of engineering, limiting their ability to effectively integrate engineering into their science instruction (Cunningham & Carlsen, 2014). This has great implications if integrated STEM is to include engineering. If opportunities for teachers to learn about integrated STEM remain scarce, teachers will continue to struggle to integrate engineering and science content without appropriate support (Roehrig et al., 2012). This is exacerbated by the fact that the nature of integrated STEM education has historically been ill-defined, leading to various definitions of what integrated STEM education is and what it can look like in the classroom (Brown, Brown, Reardon, & Merrill, 2011; Bybee, 2013; English, 2016; Herschbach, 2011; Johnson, 2012; Ring, Dare, Crotty, & Roehrig, 2017). Additionally, few studies have been dedicated to understanding how various methods of implementing integrated STEM are perceived by teachers, which is important for those wishing to support teachers new to this type of instruction.

The purpose of this phenomenological multiple case study is to provide a window into the nature of STEM education across nine teachers’ first-time implementations of STEM curricular units in middle school physical science classrooms. This study draws upon both classroom implementation data and teacher reflective interviews to not only describe different variations in implementing integrated STEM education, but to understand teachers’ challenges and successes in those approaches. This information will provide educational researchers, policy makers, and K-12 teachers with a detailed picture of what STEM integration can look like and how to support teachers who are new to STEM integration, especially when that vision of STEM integration includes engineering. The research question guiding this phenomenological multiple-case study is: *What are the commonalities and differences in nine science teachers’ experiences of implementing integrated STEM, given different variations of integrating science, mathematics, and engineering in their classrooms*?

## Literature review

### Challenges with STEM integration

#### Various models of STEM

While the number of STEM education initiatives across the country is rapidly increasing, not much is known about approaches for the implementation of integrated STEM instruction (English, 2016; Herschbach, 2011; Kelley & Knowles, 2016). This is likely due to “the limited perception of what STEM represents” (Herschbach, 2011, p. 111). For instance, Bybee (2013) offered a range of models to describe STEM education from various educational perspectives, ranging from STEM as a replacement acronym for science or mathematics to STEM as representing true integration across all four fields. Ring et al., (2017) also found that practicing science teachers conceptualized STEM integration in various ways and that these conceptions change over time as teachers write and implement integrated STEM curricula (Ring-Whalen, Dare, Roehrig, Titu, & Crotty, in press). Oftentimes, the role of mathematics within STEM has been ill-defined and in need of further investigation (Rinke, Gladstone-Brown, Kinlaw, & Cappiello, 2016; Shaughnessy, 2013). Some researchers have shied away from examining the role of technology in STEM due to the complex reality of defining technology in education (Herschbach, 2011). These various models of STEM education, however, do not necessarily provide teachers with details regarding instructional strategies that answer the question: what does integrated STEM look like in the classroom?

#### Integrated STEM in curriculum

Curriculum integration has been identified as a key component of integrated STEM (Sanders, 2009). Despite the seeming “newness” of integrated STEM education, integrated curriculum is not a new concept among educators. Practitioners and educational researchers have discussed integrating curriculum since the late 1980s as a way to increase student engagement and learning (e.g., Beane, 1991, 1995; Burrows, Ginn, Love, & Williams, 1989; Capraro & Slough, 2008; Childress, 1996; Jacobs, 1989; Sweller, 1989). Various models of curriculum integration have been identified in the literature, including models that specifically discuss science and mathematics (Davison, Miller, & Metheny, 1995); adding engineering into the mix may seem natural given recent reforms based on the technological demands of the twenty-first century (NGSS Lead States, 2013; NRC, 2012). At a curriculum level, integrated STEM has been described as integrating science, technology, engineering, and mathematics concepts in ways that reflect the practice of STEM professionals to encourage students to pursue STEM professions (Breiner, Harkness, Johnson, & Kohler, 2012). However, this seamless approach has been a difficult task for teachers who need support (English, 2016; Herschbach, 2011; Kelley & Knowles, 2016; Rinke et al., 2016), and the question of what STEM integration looks like in classroom practice remains largely unanswered.

#### A focus on engineering integration

The discipline that has dominated discussions about STEM education is engineering, as noted by national reform documents and standards (NGSS Lead States, 2013; NRC, 2012). The inclusion of engineering presents several advantages for increasing student learning (Brophy, Klein, Portsmore, & Rogers, 2008; Hirsch, Carpinelli, Kimmel, Rockland, & Bloom, 2007; Koszalka, Wu, & Davidson, 2007). A lesson or unit that includes engineering can (1) provide a real-world context to students, (2) support students’ problem-solving skills in that context, and (3) promote student communication skills and teamwork. Further, the incorporation of engineering as a context to teach science content has potential to increase both student learning and interest (Harwell et al., 2015; National Academy of Engineering & National Research Council, 2009). Despite the attention that engineering has received in K-12 education, this is still seen as an area that deserves further investigation, as imagining what STEM integration looks like in practice, especially with respect to the inclusion of engineering, has been a challenge (English, 2016; Herschbach, 2011).

Current work surrounding engineering integration in science instruction has focused on the sequencing of a given curricular unit and narrowing this sequencing into three distinct types (Crotty et al., 2017; Guzey, Ring-Whalen, Harwell, & Peralta, 2017). The first of these types aligns with engineering as an add-on in a science classroom (NRC, 2009), where a curriculum unit starts with science instruction that leads into an engineering design challenge. Engineering integration in this first type has been identified as an *add-on* or culminating project, where connections between the science content and the engineering may not be clear because of the distinct separation of engineering and science content (Crotty et al., 2017; Guzey et al., 2017). A second type that reflects the NRC’s (2009) use of engineering in integrated STEM courses has been identified as *explicit integration*, wherein a curriculum unit weaves strong connections between engineering and science content throughout the unit and students learn science through an engineering design challenge (Crotty et al., 2017; Guzey et al., 2017). A final type of engineering integration, *implicit integration*, rests in between these first two types, similar to an add-on or culminating project in which an engineering design challenge frames student learning at the beginning of the unit and is revisited at the end, but this may not necessarily connect to the science content (Crotty et al., 2017; Guzey et al., 2017). However, these types only refer to integrating engineering into science units and do not necessarily address integration that occurs among additional STEM content (e.g., mathematics) and practices.

### Teachers’ perceptions of STEM integration

The proliferation of models of STEM that exist and the associated lack of practical advice creates general confusion about integrated STEM that requires clarification, especially for teachers charged with implementing integrated STEM in classrooms (Bybee, 2010). Researchers agree that while there are many challenges, the three largest hurdles preventing successful STEM integration are (1) a lack of curriculum materials, (2) the need for creating engaging experiences for students, and (3) the need for assessments in integrated STEM (Brophy et al., 2008; Guzey, Moore, & Harwell, 2016; Moore et al., 2014; NRC, 2012; Roehrig et al., 2012; Wang, Moore, Roehrig, & Park, 2011). These three challenges are interrelated, and one might argue that, in order to address each, they must be addressed simultaneously. According to Moore et al. (2014), the role of the teacher in integrated STEM learning is to help students make abstractions and to decontextualize concepts for application in a variety of different real-world, authentic contexts. However, most teachers do not currently have the knowledge and/or equipment to bring integrated STEM to the classroom, finding the balance of developing problem-solving skills and teaching science content challenging (Dare, Ellis, & Roehrig, 2014; Wang et al., 2011). Therefore, it is necessary to understand teachers’ current beliefs, understandings, and practices of integrated STEM instruction.

This study adds to the limited knowledge of teachers’ practices and perceptions of integrated STEM. This study presents observational data from teacher participants’ curriculum implementations of integrated STEM units to illustrate how science, engineering, and mathematics are incorporated in an integrated STEM unit. This study also shares teacher perspectives on what factors, techniques, and approaches were most important to them during their integrated STEM unit. Taken together, this information will serve to clarify the nature of STEM education through teacher perspectives associated with bringing integrated STEM curriculum units to science classrooms.

### Theoretical framework

Although integrated STEM education can be modeled and defined in a number of ways, some common elements exist across current models. For the work presented here, we recognize the abundance of definitions and suggest a theoretical framework for integrated STEM education that grounds itself as, “the approach to teaching the STEM content of two or more STEM domains, bound by STEM practices within an authentic context for the purpose of connecting these subjects to enhance student learning” (Kelley & Knowles, 2016, p. 3). We expand this definition by drawing upon Moore et al. (2014) to elaborate K-12 integrated STEM education as “an effort to combine some or all of the four disciplines of science, technology, engineering, and mathematics into one class, unit or lesson that is based on connections between the subjects and real-world problems” (p. 38). These definitions highlight integrated STEM education efforts as divergent from traditional instruction with respect to both content and pedagogy. STEM content should not be taught in isolation, but rather in a way that reflects how STEM knowledge is used outside of school; this knowledge is further contextualized or driven by some problem or issue. However, there are multiple ways in which to carry this out, each of which relies on different STEM pedagogies (Brown et al., 2011; Bybee, 2013; Johnson, 2012; Vasquez et al., 2013). In order to address the problem or issue, students require the development of twenty-first century skills—creativity, critical thinking, communication, and collaboration—as they relate to the pedagogies of STEM (Bellanca & Brandt, 2010). These skills, along with adaptability, literacy, systems thinking, self-management, and self-development, have been noted as critical to STEM education (NRC, 2010).

For the work presented here, we acknowledge the specific inclusion of engineering in K-12 STEM education, which may act to contextualize and support science learning. This is driven by the STEM integration framework used in the professional development that forms the context of this study. This framework includes six major tenets for successful STEM education: (1) a motivating and engaging context, (2) the inclusion of mathematics and/or science content, (3) student-centered pedagogies, (4) an engineering design, (5) an emphasis on teamwork and communication, and (6) learning from failure through redesign (Moore et al., 2014). Together, these six tenets provide a vision of integrated STEM in which a real-world engineering design challenge contextualizes student learning of science. The purpose of this definition is to provide students with a realistic representation of how STEM knowledge is used beyond K-12 education.

By using a combination of definitions, we provide flexibility in defining integrated STEM while valuing both the integration between two or more of the disciplines and the inclusion of engineering to contextualize learning. Due to the complexity that surrounds the role of technology in STEM (Herschbach, 2011) and the emphasis on content in these definitions, we focus our exploration of STEM to the content disciplines of science, mathematics, and engineering. More importantly, this study aims to understand teachers’ experiences without imposing one specific vision of how to integrate between the disciplines.

## Methods

This study employs a phenomenological, interpretive multiple-case study design to develop an understanding of the nature of integrated STEM. This was done through an examination of middle school science teachers’ experiences with implementing integrated STEM curricular units in their classrooms, bounded by the degree to which multiple disciplines are represented during implementation (Yin, 2014). A multiple-case study design provides rich descriptions and interpretations of teachers’ experiences, and by examining multiple cases, this information provides a broader description of their experiences relating to STEM integration. The phenomenological lens used in this case study research design enabled us to better understand what implementing integrated STEM is like for these teachers, focusing on their experiences (Creswell, 2013). Phenomenology does not begin with a hypothesis about the phenomenon of study; this mitigates the influence of predetermination, presumptions, or beliefs (Sokolowski, 2000; van Manen, 1990). Because our theoretical framework makes few assumptions about the form of integrated STEM, this lens is appropriate for our use, as our goal was to “grasp the very nature” (van Manen, 1990) of STEM integration through teachers’ experiences. By using multiple cases, we were able to focus on the commonalities between integrated STEM experiences of the teachers both within and across cases (Creswell, 2013). We defined three distinct cases of STEM integration from classroom observational data. These observational data additionally provide context regarding individual classroom implementations. Post-implementation teacher reflective interviews provided detailed information from the teachers’ perspective about challenges and successes they experienced during their implementation.

### Context

This study is part of a large 5-year NSF Mathematics and Science Partnership (MSP) grant. The MSP project involves partners from higher education and K-12 schools to promote K-12 STEM integration in grades 4–8. The goal of the project is to increase student learning of science and mathematics by using an engineering design-based approach for integrated STEM instruction to guide professional development and curricular design. The STEM integration framework as described by Moore et al. (2014) was used to guide teachers’ learning during an intensive 3-week summer professional development. Specifically, the guiding paradigm of the project for STEM integration involves the integration of STEM disciplines to (1) deepen student understanding of STEM disciplines, (2) broaden student understanding through exposure to socially and culturally relevant STEM contexts, and (3) increase interest in STEM disciplines.

During the first year of the project, 48 teachers from three school districts near a large Midwestern university participated in 3 weeks of summer professional development. The professional development included partnering with instructional coaches in teams to collaborate on the creation of STEM integration curriculum units designed to address state science and mathematics standards in grades 4–8 within the context of an engineering design challenge. The summer professional development was designed to engage teachers in learning and participating in various activities as part of integrated STEM curricula. Teachers spent the first week learning about engineering design and data analysis. The second week allowed teachers to gain a deeper understanding of bringing STEM to their selected science area (life, earth, or physical science). The third week was devoted to developing and writing integrated STEM curricula. These curriculum units were expected to include the six tenets of integrated STEM education (Moore et al., 2014) discussed in the professional development. For example, teachers were asked to include an engineering design challenge situated within a real-world, engaging context that addressed science content. The PI and other project staff encouraged the use of student-centered instructional practices (such as laboratory activities and discussions) that relied on students to work in small groups. However, no prescriptive formula was provided as to how this was done (emphasizing the idea that there is no one way to write integrated STEM curricula), leading to units that varied in how STEM integration was represented, including the add-on, implicit, and explicit types of engineering integration described by Crotty et al. (2017) and Guzey et al. (2017).

## Participants

The nine teacher participants involved in this study were all middle school physical science teachers. The decision to focus on only middle school physical science teachers in this study arises from previous work (Dare et al., 2014) in which we describe physics as the science discipline best suited to readily integrate engineering. Additionally, the first author had access to all of these classrooms as their classroom coach. Of the nine participants, eight of these teachers taught 6th grade and one taught an advanced 7th grade course (Table 1). These teachers participated in three different curriculum writing groups; two of the groups focused on heat transfer in their units, and the other group focused on the particulate nature of matter (see Table 2). The nine teachers were from different school districts that represent three distinctly different student populations. Rubin School District is a large, inner-city urban school comprised of a 76% minority population with 73% of all students on free and reduced lunch. Franklin School District is an inner-ring suburban school district with a minority population of 41% and 47% of all students on the free or reduced lunch program. Noether School District is a suburban school hosting a 27% minority population with 18% of students eligible for the free or reduced lunch program. Rubin School District had just moved 6th grade from their elementary schools to the middle school setting; this meant that, in addition to being new to STEM integration, four of these teachers (Kathy, Annie, Vanessa, and Walter) were new to teaching 6th grade science in a middle school setting. With the exception of Vanessa, these teachers had previously taught in middle schools prior to this change. Ken was also new to teaching 6th grade due to a change in teaching assignment.

**Table 1:** Description of teacher participants and their co-created integrated STEM curriculum unit

	Name	Years of teaching experience	Grade	School district	Length of unit (days)	Class length (min)
*Heat Transfer 1*	Kathy^†^	6–10	6	Rubin	11	50
	Sandy	11–15	7	Rubin	14^^^	50
*Particle Nature of Matter*	Ken^†^	11–15	6	Franklin	11	53
	Tom	< 5	6	Franklin	11^^^	53
	Annie^†^	< 5	6	Rubin	13	51
*Heat Transfer 2*	Ralph	25+	6	Noether	15^^^	44
	Vanessa^†^	6–10	6	Rubin	9	65–85^‡^
	Beth	6–10	6	Noether	14	48–50^‡^
	Walter^†^	6–10	6	Rubin	16	49

All names are pseudonyms

^†^New to teaching 6th grade in 2013–2014 AY

^‡^Class periods varied according to school schedule

^^^Indicates 1 day of observation was not conducted

**Table 2:** Description of co-created integrated STEM curriculum unit

Curriculum Unit	Description
*Heat Transfer 1*	Students learn about heat transfer and density to address the engineering design challenge of creating a way to keep fish cold as they traveled from the deep ocean to the mainland in a warm climate. Students design a way to cook the fish to sell at a fish market using solar energy.
*Particle Nature of Matter*	Students focus on the importance of water. Students were engaged in a context that left them trapped in an area with only salt water and challenged to use their knowledge of the particulate nature of matter to design and build a device to desalinate water.
*Heat Transfer 2*	Students learn about heat transfer to design and build a solar oven to help children in a third-world country cheaply prepare food and sterilize water.

### Data collection

The first author observed and video recorded the implementations of the three STEM curriculum units in the 2013–2014 school year for these nine teachers as their classroom coach. In total, 111 observations were conducted; 3 days of instruction were not observed due to scheduling conflicts. Curriculum units ranged in length from 9 to 16 days (Table 1). The length of the class periods was typically around 50 min, with the exception of Vanessa, whose school kept a block schedule. Field notes were taken during each observation and used to provide a context for follow-up interviews (described below). Additionally, a digital teaching log was completed each day to indicate the length of time spent on science, mathematics, and engineering. This log included options to indicate time as follows: None, Less than 10 min, 10–20 min, 20–30 min, 30–40 min, 40–50 min, and More than 50 min.

After each teacher implemented their STEM curriculum unit, they were interviewed by the first author using a semi-structured interview protocol; this occurred at most 2 weeks after the last day of their curriculum unit implementation. This interview was structured to allow participants to reflect on their implementation, and since the first author had previously observed the STEM unit implementation, the questions were somewhat personalized to their implementation. Interviews ranged in length from 18 to 55 min.

### Defining the cases

Because of the first author’s knowledge of each classroom implementation, we knew that there were variations in how STEM integration was enacted, even across the same curriculum units. In order to capture those variations, we looked to the collected data to identify patterns, which inevitably led to us defining the cases. Knowing that the content and how it is presented in integrated STEM instruction may vary (Brown et al., 2011; Bybee, 2013; Johnson, 2012; Vasquez et al., 2013), we used information from the teaching log to create composite graphs of each teacher’s implementation that depicted how much time they dedicated to science (S), mathematics (M), and engineering (E) during each day of the implementation. This provided us with a visual representation of the nine implementations (shown in the “Results” section), which quickly confirmed that the extent of integration varied across the nine teacher participants. We further examined the extent to which one, two, or all three disciplines were represented throughout each implementation (i.e., S, E, M, SM, SE, ME, SME). Once more, we used the teaching log data to count how many days featured a single discipline and how many days were multi-disciplinary in nature (Table 3). After looking at these data, we determined that there were three broad categories that represented the degree to which integration occurred in these implementations: low (< 50% of the days included multiple disciplines), medium (50–75% of the days included multiple disciplines), and high (> 75% of the days included multiple disciplines). These cases do not align with the three curriculum units, nor do they align with years of experience. No observations included a mathematics focus, while only one observation showed a mathematics and engineering combination. As a result, the cases defined in this study correspond to these three categories, representing low, medium, and high degrees of integration. Using these boundaries helped us to better understand the differences in teachers’ experiences.

**Table 3:** Frequency of instructional days featuring single vs. multiple disciplines by case

Case	Name	S	E	Single discipline	SM	SE	ME	SME	Multiple disciplines
Low	Sandy^^^	7	2	9 69.2%	1	3	0	0	4 30.8%
	Annie	8	1	9 69.2%	1	3	0	0	4 30.8%
Medium	Ken	2	3	5 45.4%	1	5	0	0	6 54.5%
	Beth	2	4	6 42.9%	2	3	0	3	8 57.1%
	Walter	5	1	6 37.5%	3	4	0	3	10 62.5%
High	Ralph^^^	2	0	2 15.4%	2	2	1	6	11 84.6%
	Vanessa	1	0	1 11.1%	3	2	0	3	8 88.9%
	Kathy	0	1	1 9.1%	0	7	0	3	10 90.9%
	Tom^^^	1	1	2 20%	1	4	0	3	8 80%

^^^Denotes that 1 day of observation was not conducted

### Data analysis

Once these three cases were defined, we used content analysis (Miles & Huberman, 1994) to understand what factors this group of teachers felt were important to the success of STEM in their classrooms. This limited the scope of the analysis to only examine interview content that was uniquely related to STEM integration. For example, comments surrounding district-mandated time constraints were not considered. Each transcript was read and openly coded individually by the first and second authors (Corbin & Strauss, 2008). After reading and coding each interview for the given case, individual codes were discussed to check for places of agreement and disagreement between coders before moving on to reading the next transcript. Codes repeated across individual interviews allowed us to determine initial themes within each case before all transcripts were coded. Once all transcripts in all three cases were coded, we used a constant-comparative method to examine and re-examine codes to collapse them into themes across all nine interviews (Corbin & Strauss, 2008; Miles & Huberman, 1994). Once these themes were identified and refined, we further consulted the teaching logs and field notes to contextualize teachers’ comments surrounding their classroom implementation.

## Results

After analyzing the interviews as stated above, we identified three common themes across the three cases which serve to clarify the teachers’ experiences in implementing integrated STEM: *the nature of integration, choosing between science and engineering,* and *student engagement and motivation*. *The nature of integration* describes how teachers perceived their role in making connections between multiple disciplines, whether implicit or explicit. *Choosing between science and engineering* highlights the struggle that teachers felt in balancing covering science content while also engaging students in an engineering design challenge. *Student engagement and motivation* describes how teachers viewed their student’s interaction with the curriculum units they implemented. These themes, described for each case in detail below with supporting examples from classroom observations, help to shed light on the experiences that first-time implementers of STEM integration have in their classrooms. The sections that follow describe the characteristics of the case, present a visual representation of each teacher’s implementation in that case, and explore the three themes through the interviews with teachers. This section concludes with comparison and commentary across the three different cases, highlighting the stark differences and commonalities in teachers’ experience of implementing integrated STEM curriculum units.

### Case 1: Low degree of integration

**Fig. 1:**
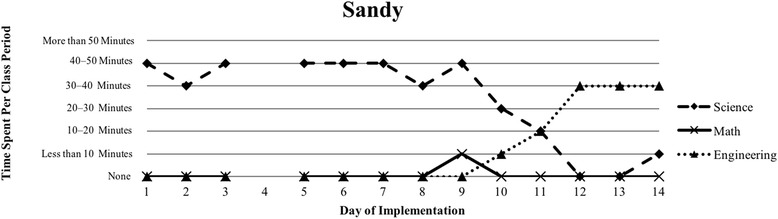
Sandy’s implementation of *Heat Transfer 1* in her 8th grade science class

**Fig. 2:**
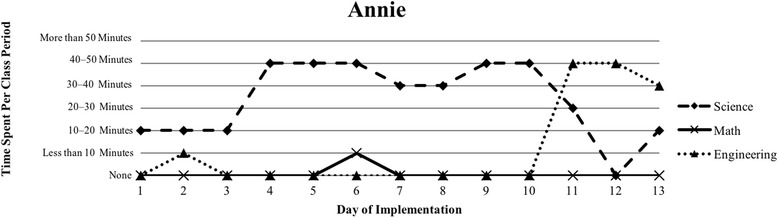
Annie’s implementation of the *Particle Nature of Matter* in her 6th grade science class

The defining feature of this first case was that less than 50% of instructional days included more than one discipline (Table 3). Additionally, the implementation by the two teachers in this case (Annie and Sandy) show a clear pattern that utilized science heavily in the beginning of the unit with more engineering towards the end of the unit (Figs. 1 and 2). Although Annie spent some time introducing the engineering design challenge on the second day of instruction, the bulk of the unit followed a pattern that is best described as science content followed by a culminating engineering project, similar to the add-on type of engineering integration (Crotty et al., 2017; Guzey et al., 2017). Table 2 displays the breakdown of how much time was dedicated to single discipline or multi-discipline class periods, revealing less integration in both teachers’ classrooms. In both implementations, more instructional time was dedicated to science, with a moderate amount of time to engineering following science instruction (Figs. 1 and 2).

#### The nature of integration

Both Sandy and Annie spent about 70% of their implementation on a single discipline with only about 30% of their time drawing upon multiple disciplines in their instruction (Table 3). In both implementations, there is a clear switch from science-focused instruction to engineering-focused instruction; this happened on day 11 for both Sandy and Annie. Mathematics instruction was rarely included in their implementations, lasting for 10 min at most and always alongside another discipline. Sandy described her experience in a way that aligned with what was observed (Fig. 1), where she consciously separated out the science content from the engineering design challenge: “It was interesting for me to teach the unit of heat and then use that as an add-on or as a way to see their—check their knowledge.” She appeared to struggle with conceptualizing what this unit would look like if she had not distinctly separated science from engineering, stating, “I wonder how it would have worked exactly if I had sort of done it all as one big unit. I don’t quite see how I could do it?” It is therefore no surprise that she favored science in her implementation.

Annie, who was positive about her implementation, shared a somewhat similar perspective regarding the complexity of teaching a STEM unit, especially when it came to teaching the specific science content (the particulate nature of matter):

I *like* the STEM component, but I think for me it was just introducing too many parts – the math and the technology and the engineering and the science – especially because I think this particular content is a little bit difficult for them [students] to understand until they get to do hands-on things where they’re seeing things.

She voiced her struggle with balancing various components of STEM in her classroom, even though she felt that it was important for her instruction and for her students. This reflects the degree to which she incorporated each discipline in her implementation (Fig. 2). Even though she spent the majority of her instructional time on science, she felt that her students struggled with the content.

Sandy and Annie felt that their students had difficulty in making connections between the science, mathematics, and engineering. For instance, Annie felt that her students failed to see connections between the science content and the engineering design challenge that asked students to create a device to desalinate water. This was most notable during a stations activity, which included such activities as dissolving salt in water and examining saturation using a PhET simulation. She commented, “I think it was too many things and that they [the students] didn’t connect. They didn’t have a flow where they’re like, ‘Oh, I can use this information from class to do this next station.’” Further, she was not confident in her students’ ability to connect to mathematics content, which might explain its limited appearance in her implementation, “I don’t think they would have got the math connection. They would have, ‘Ok, we have to measure this,’ but why? What does surface area or volume…or you know, why? Why does that make a difference?” Sandy was also not sure if her students made connections, but noted that students used scientific language during the engineering design challenge, “Um, ‘cause I did hear them using some of the vocabulary and whatnot. ‘Which one’s the best conductor? Which has the lowest specific heat?’” Because of the emphasis on science, Sandy believed that her students were successful in learning that content.

#### Choosing between science and engineering

Both Sandy and Annie essentially separated the science content from engineering until students started the design process. This was a tension for Sandy: “I thought I could do a whole bunch of stuff in a day.” For Sandy, this impacted her ability to have her students test solutions to the engineering challenge, “It was so rushed, it was really hard to test out many different solutions.” Although Sandy made sure to include time for students to complete a redesign phase so that they could learn from their initial designs, she struggled with the pressure she felt to move onto the next thing in her classroom. This was related to Annie’s feeling of having to do more work to execute a unit that was very hands-on: “I love doing the hands-on stuff, but it’s a lot more work for me, just in terms of the planning, – and they [students] love it.” Both of these teachers valued engaging their students in engineering, but recognized that it took them more time and energy to implement, which is likely why both included engineering for 3–4 days at the end of their unit.

#### Student engagement and motivation

Both Sandy and Annie had concerns about how their students engaged in the STEM unit. Sandy felt that her students were motivated and excited during this unit, noting that there was a high excitement level in her room. Simultaneously, she reflected on the fact that it was a lot of work: “And I like that, the kids liked it. It’s a lot of work in a way.” Annie was concerned about the original engineering design context of the co-written curriculum when it came time to implement, so she adjusted the context for her implementation, recognizing that she “wouldn’t have been able to sustain it [the zombie context].” Instead, she used a theme related to the reality show *Survivor* that she felt successfully engaged students. Even though she thought this was successful, she was challenged by making constant connections between what students were learning and the engineering context:

Um…kind of keeping that excitement going, I think. They were really into it and I think the *Survivor* theme helped, but keeping that in their brains. Like, “Ok, remember we’re on an island,” I think that was something that there were days that I did it really well and there were others where I mentioned it.

Annie’s method of making sure her students were reminded of the context came in the form of brief and sporadic “bellringer” activities that were very game-show-like (e.g., Jeopardy, timed puzzles). This made her think that “Overall, I think the students were engaged.” The context, to Annie, was extremely important in her instruction because “That was one thing for me and I think keeping the content exciting too because just the science part of it, it not necessarily the most exciting.” Making sure the design challenge was a real-world problem was also important, “I like having more of that real-world, like…desalination, they do it on a bigger level… but having more of that *real-world*. Like, this is, this is a real problem in the world.”

Sandy viewed engineering as, “a way to do something,” beyond just teaching the standards to her students. She viewed the addition of engineering in her classroom as, “kind of a way of teaching the science”. She noticed that some of her students were more comfortable with the open-endedness of the engineering design challenge such that they, “just took off, and the ones that weren’t kind of tried it and it was kind of fun to watch.” Sandy reflected on how the nature of her classroom changed when students were designing, becoming more engaged in the task, especially when others were being successful, “And then you’d see them going around at other groups.” It was clear that Sandy viewed the incorporation of engineering into her classroom as a way for students to communicate with one another.

### Case 2: medium degree of integration

**Fig. 3:**
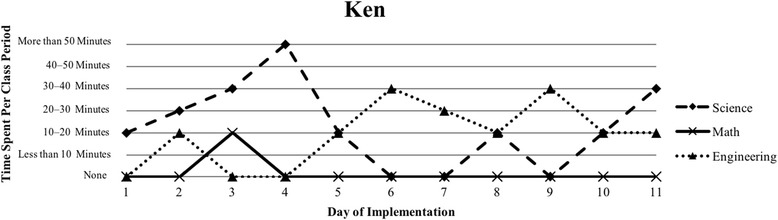
Ken’s implementation of the *Particle Nature of Matter* in his 6th grade science class

**Fig. 4:**
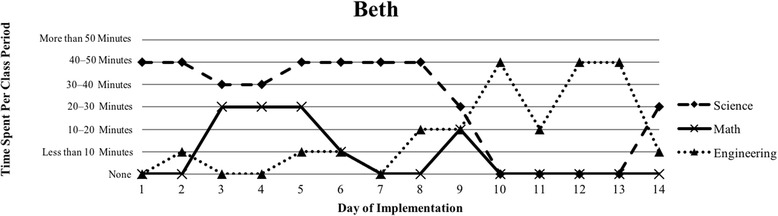
Beth’s implementation of the *Heat Transfer 2* in her 6th grade science class

**Fig. 5:**
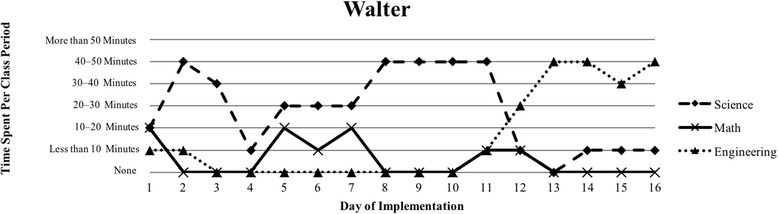
Walter’s implementation of the *Heat Transfer 2* in his 6th grade science class

The defining feature of Case 2 was that more than 50% of the days of implementation included more than one discipline, indicating a more integrated approach (Table 3). Similar to Case 1, implementations in this second case showed a pattern of more science at the beginning of the unit that transitioned into more engineering at the end of the unit (Figs. 3, 4, and 5). A total of three teachers (Ken, Beth, and Walter) comprised this case, and all three teachers introduced the engineering design challenge within the first 2 days of instruction. Most of their instruction was focused on science, but engineering and mathematics were additionally represented (Table 3). Although Ken, Beth, and Walter favored science in terms of time, they somewhat consistently drew from science, mathematics, and engineering in their implementation.

#### The nature of integration

Ken, Beth, and Walter all voiced concerns about the multi-disciplinary nature of their instruction and highlighted areas for improvement. For example, the *Heat Transfer 2* curriculum that both Beth and Walter implemented included a laboratory activity to determine what role color played in heat transfer to understand reflection and absorption. During this activity, students graphed changes in temperature over time when placing a lamp over a square piece of colored felt. Walter worried about his students’ ability to graph this information as part of the solar oven curriculum. He felt that his students were not able to transfer knowledge between graphing and interpreting data: “I just felt that they [the students] still didn’t have a good grasp of graphing and understanding the data. And even after that – did they understand – really – what they had graphed?” Walter clearly felt that his students lacked the ability to understand the mathematics and data analysis, but also did not attempt to alleviate this for his students. Ken also struggled with incorporating mathematics into his instruction, but he realized the importance of helping students make connections by talking to the mathematics teachers: “I need to work more with the math teachers too, I guess, just to make sure that we’re on the same page.” Beth had a more positive experience than either Walter or Ken, as she stressed the importance of graphing to her students early in the school year:

Graphing is also obviously a science standard, so I think I liked the graphing piece a lot. I feel like what we graphed was really easy for them [the students] to understand. But sometimes you get data that’s not so easy to graph.

After framing graphing as part of science, Beth felt her students were capable of graphing straightforward data, whereas Walter and Ken still grappled with how to do this in their classrooms. Explicitly connecting science and mathematics was important yet difficult for these science teachers who were not comfortable teaching mathematics in their classroom.

Helping students make connections between science, mathematics, and engineering was another area where teachers struggled, but also demonstrated success. For example, Beth felt positive about her students’ ability to make connections between the science and mathematics after she had her students measure the angle of reflection to understand how light behaves, stating, “I think [the angle of reflection laboratory] went really well and a lot of them [students] made the connection, ‘Oh, we’re doing angles in math class,’ so that was really good.” At the same time, though:

It was really interesting, cause I was reading through their conclusion questions and I – there was a question pertaining to, like how the angle of light affects your solar oven and how was reflection used. And not everyone could really explain it on paper.

While Beth felt that her students could make these connections in class, she was surprised that her students were not able to explain their thought processes formally. Further, outside of data analysis, Beth felt her students lacked basic mathematical reasoning when asked to scale their designs by a fourth, “Um, I was really surprised, they had a really hard time scaling down by a fourth.” This concept was not necessarily about students understanding the connections, but more about understanding basic mathematical reasoning. Similarly, Ken experienced a moment when he realized that students were not making the connections he thought were clear, “You know I guess checking for understanding along the way, but in the end it was, um, as they were building the project – that’s when I realized there was some re-teaching that had to be done.”

#### Choosing between science and engineering

Within this case, teachers noted that they wished they had given their students more time to reflect on either the science content or on a redesign for the engineering design challenge. For example, Beth recognized that her students could have benefitted from have an extra day to reflect on and redesign their solar ovens, but struggled to see where she would have had time:

I debated [whether or not to include redesign] because I really like the solar oven design, but I didn’t want to take any more days. At the same time it would be really nice to have to redesign and another day to test.

Beth’s comment addresses that redesign was important for student learning, but she felt stretched for time and obligated to move on to other topics in her room. Though Beth spent 9 days primarily teaching science or mathematics content and 5 days primarily on engineering where students were planning, building, and testing (Fig. 4), she internally struggled with balancing time to dedicate to each discipline. Ken lamented that his students, “were a little bit disappointed that it [the design process] would just end,” and “I don’t think the end results were something that I can be happy with as far as having the kids come away with being happy,” because they wanted to keep working on their designs.

Beth and Walter expressed concern in balancing how much help to give students before they embarked on their engineering design challenge. As Beth pointed out, “I don’t want to give them too many ideas so that they do it all the same way, but at the same time, maybe I should have done something so they think about, maybe how a greenhouse works.” Similarly, Walter regretted showing his students an example of a solar oven because they modeled their own designs after it and did not necessarily understand the science content that went into their designs.

#### Student engagement and motivation

Overall, these three teachers felt that that their students enjoyed the “new” way of doing science through an integrated STEM unit. Beth commented, “I mean, the kids were really excited about the solar oven,” and additionally that “…and some of the conversations – they were really good, too.” However, introducing the engineering context from the beginning proved to be challenging, even though teachers felt that it was important. For instance, Walter felt that at the beginning of the implementation, students were engaged in the context of designing solar ovens for residents of developing countries. However, Walter found that maintaining student excitement was difficult when the implementation took 3 weeks:

[The students] were really excited at first. They were like, “Alright, I can’t wait to start building,” but it took so long to get to that point. That’s where some of their frustration came in. But the excitement at the beginning – they’re like, “Alright, this sounds cool, I’m ready to go – let’s do this.”

Additionally, Ken, Tom, and Annie’s theme of survival in a zombie attack was great at engaging students at the beginning of the unit, but Ken felt this became a burden and recognized “…that trying to keep the storyline going for too long would be an issue.” He also felt that students might have been distracted by the zombie theme and did not recognize the social importance of water overuse because of the sensationalized context. “I guess I would spend as much time as possible on the phase changes and, and really just the importance of clean water. You know, socially, and how much water we use on a daily basis.”

### Case 3: high degree of integration

**Fig. 6:**
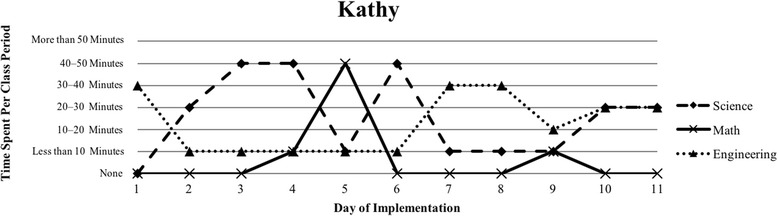
Kathy’s implementation of the *Heat Transfer 1* in her 6th grade science class

**Fig. 7:**
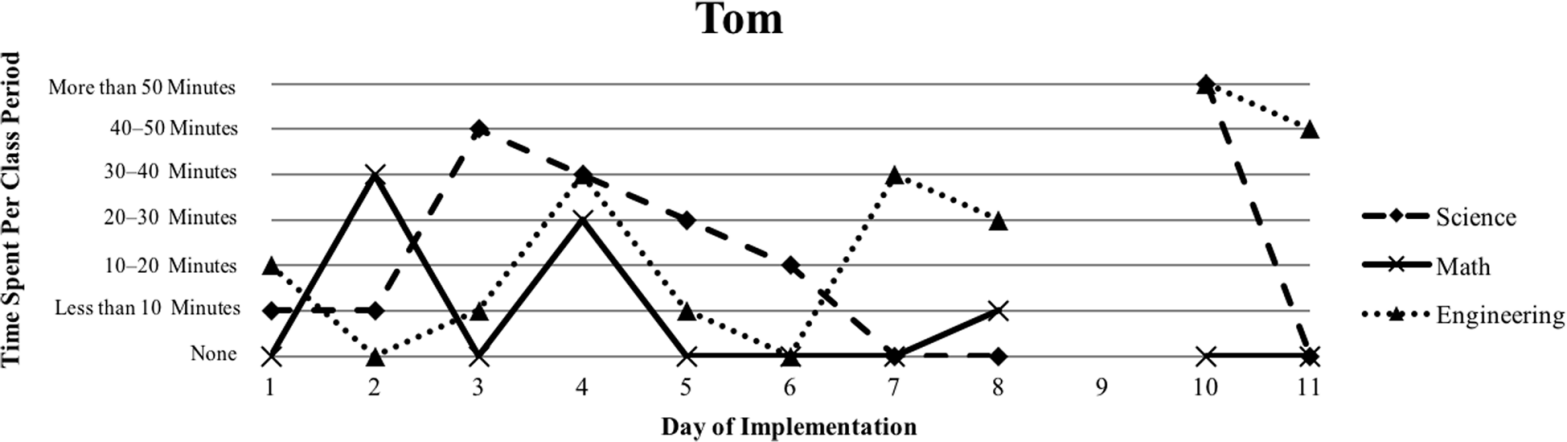
Tom’s implementation of the *Particle Nature of Matter* in his 6th grade science class

**Fig. 8:**
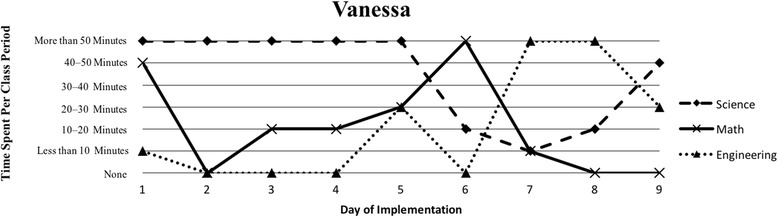
Vanessa’s implementation of the *Heat Transfer 2* in her 6th grade science class

**Fig. 9:**
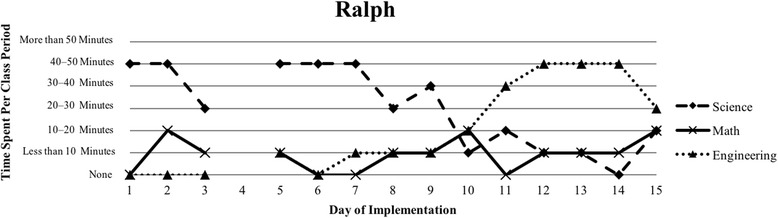
Ralph’s implementation of the *Heat Transfer 2* in his 6th grade science class

The third case consists of four teachers (Ralph, Vanessa, Kathy, and Tom) who spent more than 75% of their implementation drawing upon multiple disciplines in their instruction (Table 3). Each of their implementations wove between science, mathematics, and engineering instruction consistently throughout the unit (Figs. 6, 7, 8, and 9). Similar to Cases 1 and 2, teachers in Case 3 utilized engineering more heavily in the last few days of instruction, but also continued to weave in important science concepts that students drew upon. Vanessa, Kathy, and Tom introduced the context of their units on the first day of instruction and continued to revisit the context as students were learning the science and mathematics content needed to address their respective engineering design challenges; this is similar to explicit integration of engineering (Crotty et al., 2017; Guzey et al., 2017). While Ralph did not introduce the engineering design challenge until day 5, his implementation was one that included the most mathematics, including a day dedicated just to engineering and mathematics.

#### The nature of integration

Figures 6, 7, 8, and 9 visually represent how Ralph, Vanessa, Kathy, and Tom drew upon multiple disciplines throughout the course of their implementation. Despite the fact that all the disciplines were present, they expressed a belief that their students had difficulty making connections across the disciplines. For example, Kathy realized that her students struggled to know what materials to use as insulators because they could not connect between how an insulator worked and the materials they could choose from: “It was more about what materials they were using and understand…and having them know why they chose that material.” While students seemed to know this information, she felt that her students had trouble explaining the connection between the materials and important concepts related to heat transfer, such as insulators. She found that, “They [students] know what an insulator does…but they can’t really explain what’s actually happening to make heat transfer slow down.” This was related to her concern that

A lot of them [students] didn’t connect the whole idea of the atoms or the density to why that is, why it’s [heat transfer] slowing down. Or the thing about the air and the air bubbles, so a lot of them had trouble explaining the detail behind why an insulator works.

Because Kathy was teaching her students about insulators and heat transfer through the lens of particle density, she felt her students struggled to make connections between the science content and the engineering design challenge. She blamed this on giving students too much to do. Her thought for future implementation was that, “I think I want to expand on that [the science content] more so that I can quick make those link together better.” However, before students could obtain materials for their engineering design challenge, they had to explain to Kathy their design decisions, including justification of material choices through scientific evidence:

It let me see that they’re at least trying to pull some [science] information. Um, what we had talked about at that point in time. And when they go back out there [to their seats] they at least have some of the science understanding. And then for the redesign, the same thing, you know, when they’re listing their changes they had to justify why they were making those changes in the group and they had to agree on it and have their own ideas that they shared.

Kathy clearly made attempts at assessing student science knowledge before they could build by assessing their design plans. Although she doubted her students’ ability to make these connections, she has clearly attempted to make those connections explicit.

Tom felt similarly about his students’ understanding of the science content and their ability to connect their understanding to the design challenge: “And they [students] kind of get that salt dissolves in water, but there was a disconnect between how to actually get the salt out of water.” In thinking forward he commented, “It would be nice to figure out how to link that [the desalination process] in there somehow.” Even though he constantly drew upon multiple disciplines throughout his instruction (Fig. 7), “I feel like the science, I mean…it wasn’t…it didn’t flow as well as I wanted it to.” Further, he struggled to get his students to realize the necessity of having mathematics including in the unit:

I mean, it is hard with kids that are coming into a *science* class and like, in general, 6^th^ grade kids are going, “Ugh, it’s math,” and they kind of shut themselves down and then they’re kind of like, “This is math, why are we doing this?”

He further elaborated that while balancing the engineering, he felt the pressure to include mathematics into his instruction. He worried about how much time he had to teach mathematics content and commented, “But maybe we do have time. I don’t know, but I already feel like I’m behind the ball with putting the engineering stuff in there, or I guess the STEM unit.” Despite Tom’s critique of his own teaching, he managed to bring in multiple disciplines on a regular basis (Fig. 7).

Vanessa stressed the importance of her students’ ability to connect the science content to designing and building their solar ovens, which shows in her instruction as science and engineering are both utilized in days 7–9 (Fig. 8). It was important to her that students make these connections:

For [students] to have all that [content] information in advance and then at the end to apply it – and then to design and to build it and then to actually test it – this is the part that I spoke to them as, ‘Yeah, now I understand why we did all those labs.’ It helps to hear from them and for them to also know that, ‘Ok, this is what I need to do next because now I know about angle of reflection. I know about why it’s important to, you know, measure certain…you know, why it’s so important to graph.’

Vanessa, whose block schedule allowed for more time to develop some of the key connections between heat transfer and the engineered solar oven, made sure to take the time to guide students through this necessary reflection, which is evidenced by the fact that only 1 day of her instruction featured a single discipline (Table 3).

Similarly, Ralph felt that his students understood the relationship between the science, mathematics, and engineering after making sure these connections were explicit through laboratory activities, “because that helped them, I think, understand that their design was based on, you know, evidence.” Mid-way through his implementation, he realized that students were at a disadvantage for the design challenge because he never discussed open versus closed systems; without this information, he felt that students would not know how to design their solar oven. He ended up creating a mini-lab for students to explore the temperature effects on open versus closed solar oven designs, where students could, “collect data that informs their decision to move forward.” This additional lesson allowed Ralph to draw from science, mathematics, and the engineering design challenge to help students learn how these various concepts were all related. He was explicit in allowing students to gather data to make informed design decisions and was the only teacher to have a day dedicated to engineering and mathematics (Table 3).

Although Ralph brought in a fair amount of mathematics into his instruction, he critically reflected on the fact that making the mathematics connections explicit would have been helpful: “That’s one thing I thought…it would be nice to have them [students] write about their graph: This is what this graph is showing.” Further, Ralph offered a solution when he recognized that students struggled to connect between the disciplines, stating that students struggled to transfer mathematics content: “I’d like to have them integrate the data and graphing more with what they write about,” to better guide students to making these connections between the disciplines. Even though mathematics was included in Ralph’s implementation, allowing students time to process the mathematics would have been helpful for students to make connections between the data analysis and science content. Explicitly connecting science and mathematics was important but difficult for these science teachers who were not comfortable teaching mathematics in their classroom, and this led to them seeking help from others. For example, Ralph sought help from two of the mathematics teachers at his school to explain scaling, since, as he put it, “[students] get it when the math teacher explains it.” This was not the norm, though, as most of these teachers were unfamiliar with the mathematics teachers in their school.

#### Choosing between science and engineering

Both Kathy and Tom included a full execution of the engineering design process, including an explicit redesign phase; Vanessa and Ralph had students write about what they would do if given an opportunity to redesign. Balancing the science content and the engineering design challenge was something that teachers clearly saw as a key aspect to their success in implementing their curriculum unit. Teachers weighed the trade-off between making sure they addressed standards and making sure students understand the engineering design process. Despite this, Kathy felt, “I did move along too quickly,” and even though she thought the implementation lasted a long time, she stated, “I feel like it could have literally been a whole extra week [to teach the science content].” She elaborated:

I just felt like the last – the whole unit went on for a very long time…I had to cut it [the redesign] short so that I could get stuff in. I kind of wish I would have taken time, like, ‘Ok, let’s have each group share about what they did.’ I think I kind of rushed.

Kathy clearly felt a responsibility to her students to make sure they could learn from failure in initial designs, but felt the pull of other responsibilities (i.e., move onto the next sequence in her district-mandated curriculum guide).

Tom felt the weight of the STEM unit and struggled to add in both mathematics and engineering into his science class while still balancing teaching the science content: “I have a hard time with the amount of time we actually have to teach the science content.” He further elaborated:

I was trying to balance this engineering and then I’m also trying to think of the content that I need to teach for the trimester because we only have two trimesters with these kids, *I* have to make sure that they get these standards, and I feel like I really dropped the ball.

Although engineering standards were included in the science standards, Tom felt the responsibility of teaching the science content standards more so than having students learn about engineering.

Vanessa struggled with a similar problem in that she had dedicated too much time in making sure students were provided with the appropriate science content and not enough time on the design. “It’s just timing…I wish I had more time to redesign and test and also I think I spent so much time making sure they [students] had the [science] information that I think I took longer.” In Vanessa’s block schedule class, she dedicated 9 days to the unit in total, with 3 days dedicated to the engineering design challenge (Figure 8). Despite her weaving in engineering most days, she felt that she had to choose between science and engineering.

Ralph, who felt his students were extremely successful with their designs, also felt he rushed his students through the design process, stating, “I think it was just going too fast for them to process sometimes.” He felt that each class was a whirlwind of getting materials, building, and cleaning up, leaving little time for students to stop and think about what they were doing. He wished that he had “more time to reflect to try to bring it back to the client again.”

#### Student engagement and motivation

Kathy, Tom, Vanessa, and Ralph were all optimistic that their students were engaged in their STEM unit. Kathy commented, “I would say all of my classes were generally engaged in making the freezer…They were into making it.” She felt that the unit, “…engages them more to be honest. It is more hands on.” Specifically, Kathy noted that the inclusion of a budget was oddly satisfying to her students where, as she thinks about future implementation: “I’m thinking about getting paper money for them next year because they were so excited about that. Um, and the whole, like refund…they’re just so funny with it, you know, ‘I want a refund’ or ‘Is this on sale?’” She focused on the fact that her students were interested in this and wanted to encourage their participation.

Tom felt that, overall, “I think our engineering design challenge is good. And I…I mean, they [the students] like it…and I like that part.” However, though he enjoyed the context and thought that students were into it, he struggled with it:

I liked the context and I liked being able to get them involved in it I think throughout, like just, I mean…kids are into that zombie stuff, but I mean just pulling something big and like kind of keeping that and I know that there were times where I dropped the ball in like bringing that context into it, but I think for the most part they were really excited to do it. They were really engaged in the overall context of it.

Tom was cognizant of the fact that the context was what drove his students to be excited, but he personally struggled with bringing it into each lesson. Though Tom was not necessarily positive about the connections made between science and engineering, he commented, “I like the conversations that went on – the engineering conversations that went on.” He noted that, “a lot of groups did a really good job of working together and talking about engineering ideas and how each one contributed a little here, there and…I felt that was good.” He felt the need for this unit to enable students to learn from one another through discussion of design decisions:

I heard a lot of good conversations with that and walking and just kind of checking in with groups, ‘Why are you doing this,’ and just the way that some of the groups were working together in a way that you know, they would bounce back…bounce ideas back and forth off of each other. I thought that was successful.

Vanessa and Ralph commented similarly, specifically pointing out the energy in the room and the collaborative elements that were incorporated into the unit they constructed. Vanessa noted the excitement that students had: “And they have fun. The kids really, really had fun.” She elaborated on this to note that she was, “excited them and gave them a purpose and a reason to do it [the unit].” Ralph offered that this was a great way for students to collaborate with one another. “Um, so…and I, you know, it was a great group assignment. It was…all that was great. I mean the kids were very excited about it. It [the solar ovens] actually worked.”

### Cross-case findings

Across the three cases, there were similarities and differences with regard to the identified themes.

#### The varied nature of integration

Our interviews revealed that all teachers struggled to balance addressing each of the three STEM disciplines, with many teachers explicitly indicating their uncertainty about integrating between the subjects. This reflects the patterns seen in Figs. 1, 2, 3, 4, 5, 6, 7, and 9, where there are clear areas where one discipline is favored over another within the implementation. This is most prominent in Case 1, where both Sandy and Annie reflected on the difficulty in adding in the “extras” of engineering and mathematics to their science instruction. Despite the inclusion of more mathematics and engineering in Case 2, Ken, Beth, and Walter struggled to support students’ understanding of graphing and data analysis, which impacted students’ understanding of the science content and engineering design solutions. These teachers were most concerned about students’ inability to collect and graph data and interpret graphical information; Beth also felt that her students struggled with mathematical reasoning. In Case 3, Kathy, Tom, Vanessa, and Ralph strived to make sure that the science content and the engineering design challenge were planned well enough for the connections between those disciplines to be obvious to their students. While Kathy, Tom, and Vanessa were also concerned about their students’ mathematical abilities in connection to the science content, only Ralph was explicitly concerned about the need to make clear connections between data analysis and engineering design decisions.

All teachers were concerned about the ways in which students were able to connect between the science content, mathematics content, and the engineering design challenge. Despite the emphasis in the professional development on helping teachers find ways to connect between STEM content disciplines, our evidence suggests that these teachers still struggled with integrating mathematics and engineering into their science instruction. However, this struggle appeared to be somewhat alleviated when teachers made explicit connections between these disciplines.

#### Science versus engineering: a false dichotomy?

Although these curriculum units were intended to include a full execution of an engineering design process, only Kathy, Tom, and Sandy included an explicit redesign phase. Most teachers felt that balancing the roles of each piece of STEM was challenging with respect to time. Teachers from Case 1 favored science, whereas teachers from Cases 2 and 3 presented a version of STEM that, while still science-heavy, somewhat consistently brought in aspects of either engineering or mathematics. Interviews from all cases additionally revealed that teachers viewed teaching science content and dedicating time to engineering design or redesign as a balancing act. This separation of the two areas emphasizes the idea that teachers were novices at integrating them and struggled with making sure their students had time to process the information and complete their assigned tasks.

#### Maintaining student engagement

Across all three cases, teachers believed that their students not only benefitted from learning about engineering, but were engaged in the overall integrated STEM curriculum. Despite some of these challenges, teachers felt that the high percentage of hands-on activities included in their curricula was the reason for success in the classroom. This was not limited to just the engineering design challenge, but extended to the importance of student learning through hands-on laboratories. Although an engaging context was important to keep students motivated throughout the unit, maintaining this context over the course of up to 3 weeks was challenging for a number of teachers. Apart from Ralph and Sandy, teachers in all three cases started the curriculum unit by introducing this context on the first or second day of instruction as a way to motivate student learning of the science content. Both Ralph and Sandy introduced the engineering design challenge after the bulk of science content had been delivered, but neither noted if this was an advantage. This meant that for roughly 3 weeks, teachers not only taught the relevant science content, but also attempted to maintain student engagement by utilizing the engineering design context.

## Discussion

### Silos: a mental model?

Our findings shed light on how middle school physical science teachers implement and experience an integrated STEM curriculum for the first time. While the themes presented above represent both successes and challenges faced by these teachers, they also illuminate tensions that exist regarding successful STEM integration. Our teachers felt the need to incorporate and balance the STEM disciplines, but struggled to find the “time” to do this. In many instances, this was due to the teachers still teaching these concepts separately, choosing to engage in add-on engineering integration as opposed to explicit integration (Crotty et al., 2017; Guzey et al., 2017). Our teachers clearly wanted their students to know the relevant mathematics and science content and apply that content to both laboratory activities and engineering design challenges, but they struggled to do this in a way that did not take up too much time. While physical science provides a natural avenue for students to apply mathematical knowledge in a science classroom, our teachers felt that their students were unable to “do math” in their science classrooms. This idea of science teachers failing to include computational thinking in their instruction has been observed in the literature (Rinke et al., 2016) and is a clear place to improve upon when it comes to integrated STEM instruction.

One possible explanation for their discomfort with teaching mathematics related to engineering is that our teachers were science teachers and not mathematics teachers or engineers; they struggled with how to use the design challenge to teach the necessary mathematics content. This may be why Ralph asked the mathematics teachers at his school to make a video about scaling, which helped his students make direct connections between the science and mathematics content. The video introduced the mathematics concepts with practical and relevant connections to Ralph’s solar oven engineering design challenge that Ralph’s students needed to be successful; in essence, the engineering provided students with a relevant and explicit context for learning the mathematics content. With the exception of Ralph, the other teacher participants generally avoided incorporating mathematics to any great detail and felt that their students struggled to do anything more than basic data analysis. These experiences are in line with other literature that suggests that teachers cannot assume that students will see the mathematics connections; rather, these connections must be “transparent and explicit” (Shaughnessy, 2013, p. 324). Helping students make these connections is an area of research that is still in need of further exploration (English, 2016).

One of the major challenges for these teachers was maintaining a balance between teaching the science content that they were required to teach and making sure the engineering design challenge was (1) engaging to students and (2) something that their students could reasonably do. This was present throughout all cases. It is clear from the interviews that these teachers, overall, still viewed science content and engineering design/redesign as two separate entities. Even in Case 3, mathematics and engineering were seen as add-ons to their science classes, despite the fact that teachers felt the need to make these connections for their students. While teachers saw the need for students to make connections between the disciplines, the execution of the curriculum prevented students from doing this, since the typical pattern was science and mathematics content first, followed by the engineering design challenge. Even if the context was presented on the first day and revisited throughout the implementation (as seen in Case 3), these teachers felt that they still were not explicit enough. This struggle could be related to the teachers’ inexperience in teaching more than science content in their classrooms.

### Keeping it real for students

The creation of real-world, meaningful contexts was emphasized in both the professional development and the STEM integration framework (Moore et al., 2014) and was seen by teachers as important to their success. However, these teachers struggled with maintaining an interesting and realistic enough storyline for their students to keep interest. This feature is rather unique to integrating engineering in K-12 instruction and therefore challenged these teachers to consider how science is used in the real world.

In this work, the introduction of a STEM-integrated unit to science teachers signals a significant shift in the *status quo* of the physical science classroom, and we believe that this shift causes insecurity with even the most experienced teachers. These teachers were compelled to evaluate how they balance teaching science content, teaching mathematics content, guiding students through an engineering design challenge, and integrating between these areas. In future integrated STEM instruction, we believe that teachers must move away from choosing between content and engineering and must be better supported in finding opportunities to truly integrate the STEM disciplines by leveraging science content through an engineering design challenge.

## Conclusions

This work provides a window into what integrated STEM education can look like in practice within middle school physical science classrooms, focusing on more than just the sequencing of engineering within a STEM unit (Crotty et al., 2017; Guzey et al., 2017). Our findings indicate that there are differing degrees of integration when it comes to an integrated STEM approach. Further, it appears that the degree of integration may be related to teachers’ awareness of how to make explicit and meaningful connections between the disciplines. If teachers find such integration valuable, they may be more willing to spend the time helping their students make those connections. Teachers in this project were supported throughout their implementation, but it is unlikely that this can happen in every case where teachers are being asked to engage in integrated STEM instruction in their classrooms. Our findings suggest that teachers need continued support as they navigate bringing multiple disciplines into their classrooms. Those who value making explicit connections, like the teachers in Case 3, will likely continue to constantly interweave multiple disciplines in their instruction on a regular basis.

### Limitations and future work

The findings presented here indicate to educational researchers, policy makers, and K-12 STEM educators that there are several challenges when it comes to implementing new STEM initiatives in K-12 education. Although this work is limited to middle school physical science teachers’ experiences with first-time STEM instruction, many of the identified themes are not content-specific; therefore, this work may shed light on general struggles that are common across subject areas when introducing integrated instruction for the first time. Future work could determine the role that content—as well as other factors such as grade level—may play in the degree of integration.

While this phenomenological work is composed of teacher reflections on their experiences with first-time STEM implementation, we are interested in continuing to explore how these reflections relate to their teaching pedagogy during this implementation. By making use of quantitative observational measures throughout the entire curriculum unit, we could construct a picture of each teachers’ evolving pedagogy and instruction as they implement a STEM integrated unit for the first time. Currently, quantitatively assessing the quality of integrated STEM education in K-12 classrooms is challenging due to the lack of available observational tools that capture the varied essence of integrated STEM. Understanding the ways in which teachers integrate engineering into their science instruction is a necessary first step in considering approaches to assessing the quality of integrated STEM instruction.

While this study did not attempt to assess the quality of the instruction, our findings provide examples of three ways in which teachers implement integrated STEM into a unit of instruction. Further study is required to understand how the degree of integration relates to the quality of instruction and also to student learning outcomes. The findings here suggest that teachers who engage in larger amounts of integrated instruction value providing an authentic learning context that makes clear connections between disciplines, reflecting key components of integrated STEM education (Moore et al., 2014; Kelly & Knowles, 2016). While we have provided information about the amount of time dedicated to science, engineering, and mathematics, assessing the quality of this instruction could not only substantiate teacher reflections on their own teaching, but could benefit administrators and evaluators in helping them understand the needs of teachers who bring STEM to their classrooms. Until that time, however, this current study can act as a resource to help future facilitators and leaders of professional development to understand the challenges teachers may face when bringing STEM to their classrooms.
